# Hwang-Heuk-San induces apoptosis in HCT116 human colorectal cancer cells through the ROS-mediated activation of caspases and the inactivation of the PI3K/Akt signaling pathway

**DOI:** 10.3892/or.2022.8301

**Published:** 2022-03-10

**Authors:** Moon Hee Lee, Su-Hyun Hong, Cheol Park, Gi-Young Kim, Sun-Hee Leem, Sung Hyun Choi, Young-Sam Keum, Jin Won Hyun, Taeg Kyu Kwon, Sang Hoon Hong, Yung Hyun Choi

Oncol Rep 36: 205–214, 2016; DOI: 10.3892/or.2016.4812

Subsequently to the publication of the above article, an interested reader drew to the authors attention that certain of the data panels featured in Figs. 1B, 4A, 6A and 8A, showing DAPI or NAC staining of the cells, appeared to contain overlapping data. The authors have consulted their original data, and realize that errors were made during the compilation of these figures; consequently, they have repeated the affected experiments.

The revised versions of Figs. 1, 4, 6 and 8, featuring replacement data for Figs. 1B, 4A, 6A and 8A, are shown on the subsequent pages. The authors regret the errors that were made during the preparation of the published figures, and confirm that these errors did not affect the conclusions reported in the study. The authors are grateful to the Editor of *Oncology Reports* for allowing them the opportunity to publish a Corrigendum, and all the authors agree to this Corrigendum. Furthermore, they apologize to the readership for any inconvenience caused.

## Figures and Tables

**Figure 1.**
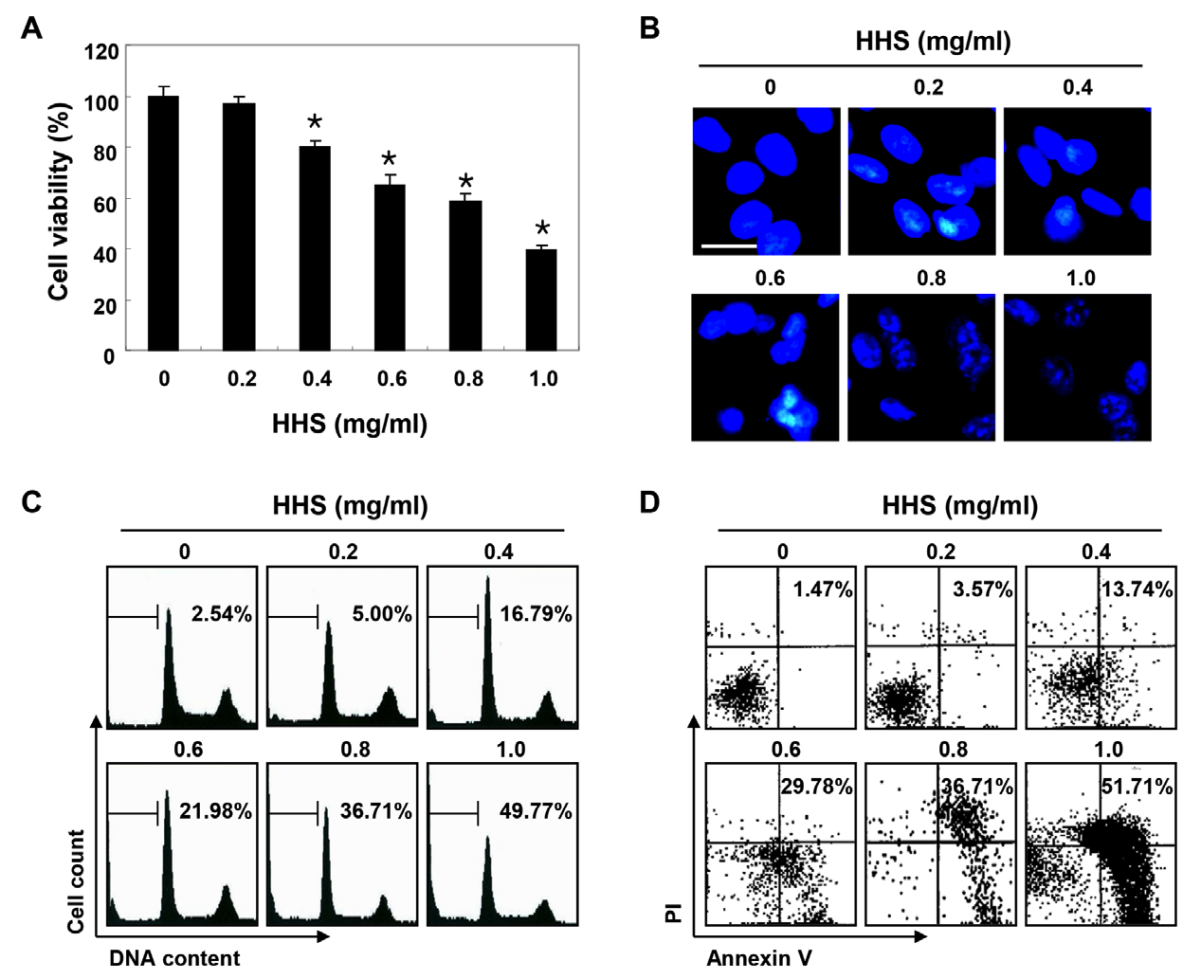
Inhibition of cell viability and induction of apoptosis by HHS treatment in HCT116 cells. (A) The cells were treated with various concentrations of HHS for 48 h. Cell viability was determined by the MTT assay. Statistical significance was determined using one-way ANOVA (*p<0.05 vs. untreated control). (B) The cells grown under the same conditions as (A) were fixed and stained with DAPI to visualize DNA. The stained nuclei were then observed under a fluorescence microscope using a blue filter (original magnification, ×400). (C) To quantify the degree of apoptosis induced by HHS, the cells were evaluated by a flow cytometer to determine sub-G1 DNA content, which represents the cells undergoing apoptotic DNA degradation. (D) The cells were also stained with Annexin V-FITC and PI, and the percentages of apoptotic cells (Annexin V+ cells) were then analyzed using flow cytometric analysis. (C and D) The results are expressed as the mean of two different experiments.

**Figure 4.**
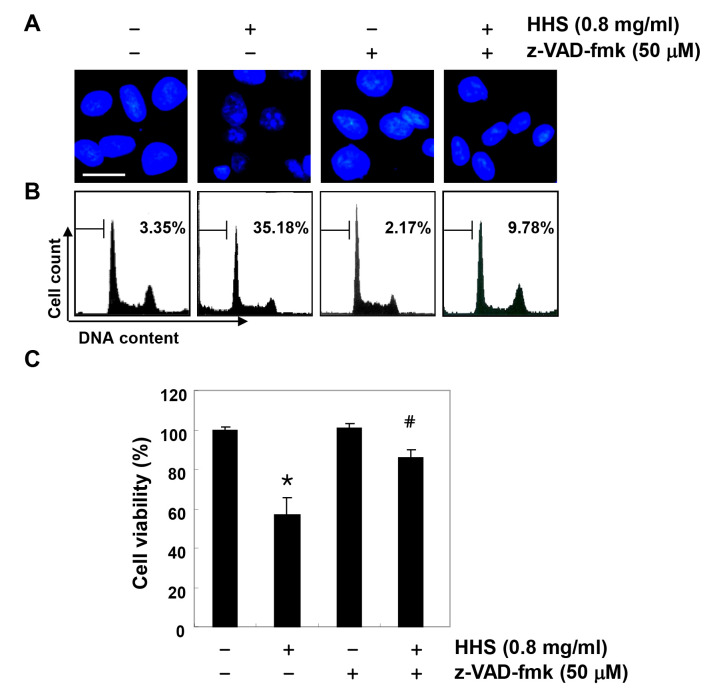
Inhibition of HHS-induced apoptosis by pan-caspase inhibitor in HCT116 cells. The cells were pre-treated for 1 h with or without z-VAD-fmk (50 μM) and then treated with HHS (0.8 mg/ml) for an additional 48 h. (A) The cells were stained with DAPI and photographed with a fluorescence microscope (original magnification, ×400). (B) The percentages of apoptotic cells (sub-G1 cells) were analyzed using flow cytometric analysis. The results are expressed as the mean of the two different experiments. (C) Cell viability was determined by the MTT assay. Each point represents the mean ± SD of three independent experiments (*p<0.05 vs. the untreated control; ^#^p<0.05 vs. the HHS-treated cells).

**Figure 6.**
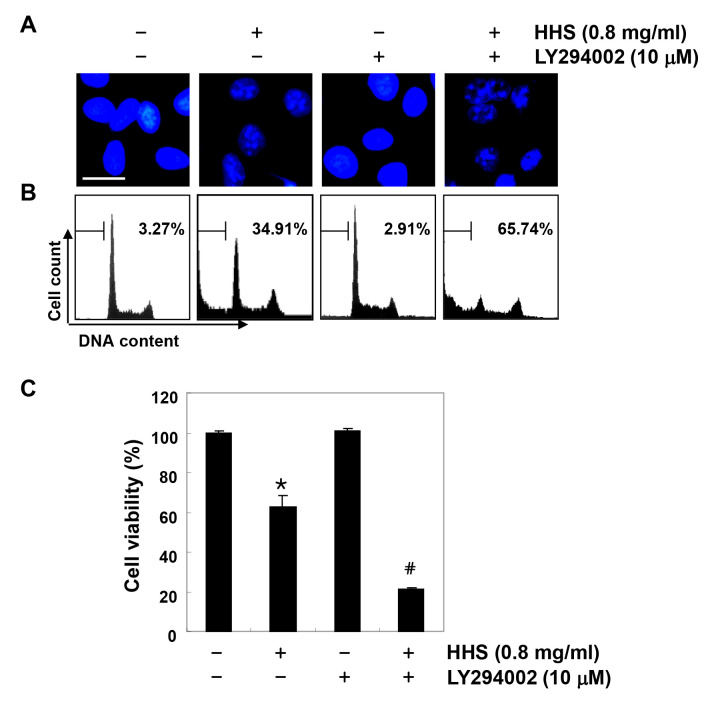
HHS-induced apoptosis is associated with the inactivation of PI3K/Akt signaling in HCT116 cells. The cells were pretreated with PI3K inhibitor (LY294002, 10 μM) for 1 h and then treated with HHS (0.8 mg/ml) for 48 h. (A) After staining with DAPI solution, the nuclei were observed under a fluorescence microscope (original magnification, ×400). (B) The percentages of apoptotic cells (sub-G1 cells) were analyzed using flow cytometric analysis. The results are expressed as the mean of the two different experiments. (C) Cell viability was determined by the MTT assay. Each point represents the mean ± SD of three independent experiments (*p<0.05 vs. the untreated control; ^#^p<0.05 vs. the HHS-treated cells).

**Figure 8.**
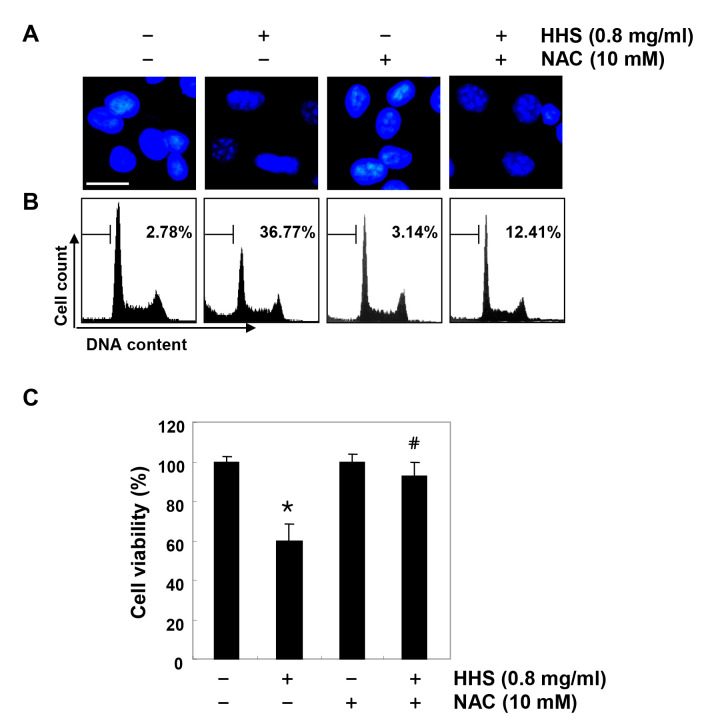
ROS-dependent apoptosis induction by HHS treatment in HCT116 cells. (A) The cells were treated with or without NAC (10 mM) for 1 h before challenge with HHS (0.8 mg/ml) for 48 h. (A) They were collected and stained with DAPI to visualize the DNA. The stained nuclei were then observed under a fluorescence microscope (original magnification, ×400). (B) To quantify the degree of apoptosis, the cells were evaluated for sub-G1 DNA content using a flow cytometry. The results are expressed as the mean of the two different experiments. (C) Cell viability was determined by the MTT assay. Each point represents the mean ± SD of three independent experiments (*p<0.05 vs. the untreated control; ^#^p<0.05 vs. the HHS-treated cells).

